# Reply: Sodium-Glucose Cotransporter 2 Inhibitor Use and Outcomes in Transthyretin Amyloid Cardiomyopathy

**DOI:** 10.1016/j.jacadv.2025.101696

**Published:** 2025-04-25

**Authors:** Vikash Jaiswal, Muhammad Hanif, Yusra Mashkoor, Tanisha Prasad, Gregg C. Fonarow

**Affiliations:** a Larkin Community Hospital, South Miami, Florida, USA; b SUNY Upstate Medical University, Syracuse, New York, USA; c Dow University of Health Sciences, Karachi, Pakistan; d Royal College of Surgeons, Dublin, Ireland; e Ahmanson-UCLA Cardiomyopathy Center, Ronald Reagan UCLA Medical Center, Los Angeles, California, USA

We appreciate the insightful comments and suggestions from Dr Karakasis regarding our study titled, “Sodium-Glucose Cotransporter 2 Inhibitor Use and Outcomes in Transthyretin Amyloid Cardiomyopathy.”^1^ These observations provide valuable perspectives on the interpretation of our findings.

As this was a retrospective study conducted using the TriNetX database, we faced inherent limitations, including the lack of detailed information on disease stages, which we acknowledge as a major limitation. While we attempted to address disease-specific nuances, the unavailability of such data restricted our ability to evaluate this aspect comprehensively.^2^ While these limitations were briefly mentioned in our paper, the limited word count due to a short report format restricted us from discussing them in greater detail.

Regarding the matching of tafamidis use, we agree that it was not ideal, though the standardized difference was 0.06. Nevertheless, we acknowledge that imbalances in tafamidis use could have influenced the results, and this remains an area requiring further exploration.

We also concur with Dr Karakasis that the lack of significant effects on heart failure, atrial fibrillation, and ventricular tachycardia in our study necessitates critical interpretation. Transthyretin amyloid cardiomyopathy pathophysiology, involving myocardial infiltration and high filling pressures, is unlikely to be fully addressed by sodium-glucose cotransporter 2 (SGLT2) inhibitors alone. However, these agents may confer systemic benefits, such as improved vascular function and reduced inflammation, which could contribute to the modest reductions observed in major adverse cardiovascular events and ischemic stroke.^3^ We also matched for left ventricular ejection fraction to address confounding related to heart failure outcomes, but further research is needed to determine whether the benefits of SGLT2 inhibitors are disease-modifying or adjunctive.

As noted, the findings from our study are not generalizable to other forms of amyloidosis. We agree that large, randomized clinical trials are essential to evaluate SGLT2 inhibitors across various types of amyloidosis, focusing on disease severity and other critical parameters. Such studies will provide a deeper understanding of these drugs' efficacy and safety in this complex patient population.

We thank Dr Karakasis for their constructive feedback, which has further clarified the study’s strengths and limitations. We hope that this exchange will encourage more focused research on the role of SGLT2 inhibitors in transthyretin amyloid cardiomyopathy.
